# Interplay Between Genetic Diversity and Tree Vitality in *Fraxinus excelsior* Populations Affected by Ash Dieback

**DOI:** 10.3390/genes16091087

**Published:** 2025-09-16

**Authors:** Justyna Nowakowska, Jakub Słowik, Artur Pacia, Anna Tereba, Aleh Marozau, Piotr Borowik, Tomasz Oszako

**Affiliations:** 1 Faculty of Biology and Environmental Sciences, Institute of Biological Sciences, Cardinal Stefan Wyszynski University, Wóycickiego 1/3, 01-938 Warsaw, Poland; j.nowakowska@uksw.edu.pl; 2 Faculty of Civil Engineering and Environmental Sciences, Institute of Forest Sciences, Białystok University of Technology Białystok, ul. Wiejska 45E, 15-351 Białystok, Polanda.marozau@pb.edu.pl (A.M.); 3 Chojnów Forest Inspectorate, Pilawa, Klonowa 13, 05-532 Baniocha, Poland; artur.pacia72@gmail.com; 4 Forest Silviculture Department, Forest Research Institute, ul. Braci Leśnej 3, 05-090 Sękocin Stary, Poland; a.tereba@ibles.waw.pl; 5 Forest Protection Department, Forest Research Institute, ul. Braci Leśnej 3, 05-090 Sękocin Stary, Poland; pborow@poczta.onet.pl

**Keywords:** *Hymenoscyphus fraxineus*, inbreeding coefficient, selective breeding, Roloff vitality classification, forest pathology, genetic resilience, conservation genetics

## Abstract

**Background:** Ash dieback, driven by the invasive fungal pathogen *Hymenoscyphus fraxineus*, has precipitated severe declines in *Fraxinus excelsior* L. populations across Europe, threatening genetic diversity and ecosystem stability. **Methods:** This study investigates the interplay between phenotypic vitality and genetic variation in five Polish ash stands using nuclear simple sequence repeat (nSSR) and chloroplast DNA (cpDNA) markers. Vitality assessments of 186 trees across three reserves (from Białowieża Primeval Forest and Wolica Reserve) were conducted. **Results:** Vitality assessments revealed a slight predominance of dying individuals (36%, 3rd degree of Roloff classification). Nuclear analyses indicated moderate to high diversity (mean *H*_E_ = 0.826), significant inbreeding (*F*_IS_ = 0.178, *p* < 0.001), and low inter-population differentiation (*F*_ST_ = 0.044) among all five stands. Chloroplast markers showed elevated differentiation (Φ_ST_ = 0.228, *p* < 0.0001), reflecting phylogeographic structure. Vitality degrees assessed in three chosen populations (Browsk FD, Hajnówka FD, and Chojnów FD) exhibited negligible genetic differentiation (nSSR *F*_ST_ = 0.009; cpDNA Φ_ST_ = 0.003), suggesting gene flow mitigates pathogen-induced selection. Bayesian clustering (STRUCTURE, K = 3) revealed admixture with distinct genotypes in dying trees, potentially linked to susceptibility. **Conclusions:** These findings underscore the resilience of ash genetic pools and advocate for selective breeding in nurseries to prevent the spread of dieback, prioritizing resistant genotypes for conservation.

## 1. Introduction

Forest ecosystems composed of diverse tree species, including European ash (*Fraxinus excelsior* L.), play a crucial role in maintaining ecological balance, supporting biodiversity, stabilizing microclimates, and enhancing adaptability to environmental changes, particularly in response to biotic stressors such as pathogen invasions. Since the 1990s, ash dieback, caused by the ascomycete *Hymenoscyphus fraxineus* (T. Kowalski) Baral, Queloz, and Hosoya (formerly *H. pseudoalbidus*, introduced from East Asia), has led to severe crown defoliation, vascular necrosis, and mortality rates often exceeding 80% in affected populations [1,2,3].

Acting as an endophyte, the pathogen infects leaves, causing necrotic lesions, wilting, and ultimately crown dieback. Mortality rates reach up to 70% in natural forests and 85% in plantations [4]. The disease leads to extensive shoot necroses and branch desiccation, which can be classified using vitality degrees ranging from weakened (I) to dead trees (IV) [5].

Common Ash Dieback (ADB) was first documented in Poland (Czerwony Dwór) in 1992 [1] and has since spread across Europe, affecting most of the natural range of *F. excelsior*. The disease is most severe in humid regions with moderate temperatures, where the pathogen produces apothecia on fallen leaves and releases spores [6]. In Belarus, ADB prevalence in mature stands reaches 90%, with an average branch affliction of 42.9 ± 4.2%. Second-layer trees show 73.3% prevalence, undergrowth from seeds 14.8 ± 4.2%, and stump shoots are consistently affected [1].

Given that adaptive responses primarily occur in natural populations, monitoring tree health in forest reserves is of high scientific interest. In Poland and Belarus, ash stands in protected areas such as the Białowieża Primeval Forest (BPF) face compounded threats from habitat fragmentation and climate change. In the Belarusian section of BPF, ash mortality rose from 6.8% in 2003 to 12.2% in 2004, far exceeding the baseline annual rate of 1.3%. Over 80% of stands were classified as resilience-impaired, with only 12% considered biologically sustainable [7]. This alarming decline was also monitored through the National Forest Monitoring Network established in 2003, and further addressed within the framework of Project 2006 [8,9]. By 2005, projections indicated near-total loss of ash within 2–3 years [10].

Comparative vitality assessments revealed ash’s low viability (III.1-IV.3) relative to maple (I.4-II.7) and linden (I.5-II.8), with *H. fraxineus*-induced necrosis exacerbated by *Armillaria* spp. and stem pests. Other studies confirmed that 90% of mature ash trees in Belarus exhibited branch affliction averaging 42.9 ± 4.2%, primarily due to *H. fraxineus*, verified via PCR and Sanger sequencing. Forest stand transformation toward maple-hornbeam assemblages was accelerated, while ash regeneration was suppressed by ungulate browsing pressure [11].

By contrast, Polish Białowieża Primeval Forest (BPF) lacks detailed dieback records, highlighting the need for genetic studies. Population genetics may reveal adaptive mechanisms to biotic stressors, as invasive pathogens can intensify genetic erosion in fragmented habitats [12].

The pace of contemporary environmental change poses a significant challenge for *F. excelsior* populations affected by the ADB phenomenon. Several questions arise in this context, including knowledge about the ash genome and its structural and functional properties, which could lead to the species’ adaptive processes under ongoing climate change [13,14,15]. The nuclear microsatellite (nSSR) and chloroplast (cpDNA) markers are pivotal for understanding the genetic basis of tree health status in the face of environmental stresses and pathogens. In *F. excelsior* stands, genetic markers including nSSR, cpDNA, and genome single-nucleotide polymorphism have been used to identify correlations with tolerance to decline caused by *H. fraxineus* [16,17,18,19,20,21]. The molecular approach can lead to the application of markers for selecting genotypes resistant to pathogens in other deciduous tree species, among *Quercus* sp. and *Fagus* sp. For instance, analysis of nSSR and cpSSR in *Quercus infectoria* (Oliv.) revealed a correlation between genetic diversity and tree health in Iran, where lower cpDNA diversity may indicate higher susceptibility to degradation [22]. Analysis of sympatric *Quercus* species (*Quercus petraea* (Matt.) Liebl., *Q. pubescens* Willd., and *Q. frainetto* Ten.) using nSSR showed a correlation between genetic diversity and crown health status in Serbia [23]. Comparable studies were conducted for *Fagus sylvatica* (L.) in the aim of assessing genetic diversity in the context of adaptation to environmental conditions such as drought or pathogens affecting tree vitality [24,25].

In our work, we focused on identifying correlations between the level of crown damage in three common ash populations, including two located in the BPF (eastern Poland) and one in the Nature Reserve in central Poland, and the level of genetic diversity at the level of selected nuclear and chloroplast DNA markers. This study addresses gaps in linking the vitality of trees from protected areas to genetic parameters using nSSR and cpDNA markers, hypothesizing that weakened or dying trees exhibit reduced heterozygosity and increased inbreeding due to pathogen selection, while private alleles signal adaptive potential.

## 2. Materials and Methods

### 2.1. Study Area and Sampling Design

**Figure 1:**
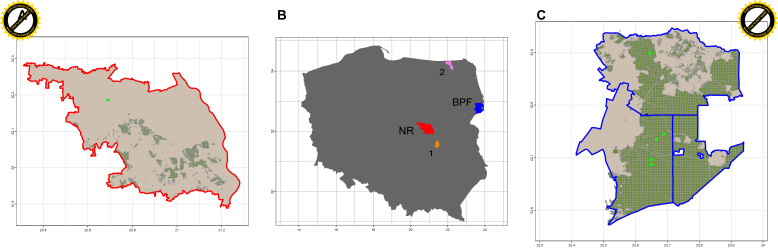
Geographical distribution of studied European ash (*F. excelsior*) stands. Central panel (**B**)—location of all studied populations: Nature Reserve (NR); Chojnów FD (central Poland, (**A**) panel); Browsk and Hajnówka FDs within the Białowieża Primeval Forest (eastern Poland, (**C**) panel). Two reference populations: 1—Kozienice FD; 2—Czerwony Dwór FD.

The main visible symptoms include wilting and dieback of the crown, leaf discoloration and necrosis, lesions on shoots and branches, and characteristic blackened basal parts of the leaf petioles. Progression of symptoms often leads to gradual decline in tree vitality and eventual mortality. To ensure the specificity of our assessments, we explicitly excluded trees showing signs of weakening due to other common stressors, such as root rot caused by *Armillaria* species, stem rots, bacterial infections, and damage from bark beetle infestations. This careful differentiation helps isolate the effects of ash dieback disease on the studied populations. Five European ash (*F. excelsior*) populations were sampled in 2016 from Polish forest stands selected for varying fragmentation and dieback prevalence. For the vitality degree assessment, three field screenings were conducted in forest stands dominated by *F. excelsior* within the Białowieża Primeval Forest (BPF)—specifically in the Browsk and Hajnówka Forest Districts (FD)—and in the Wolica Nature Reserve within the Chojnów Forest District (Table 1). According to the natural-forest regionalization of Poland, the BPF area is situated within the Mazurian-Podlasie Land II in eastern Poland, corresponding to the Białowieża Primeval Forest mesoregion (Figure 1).

On the other hand, the Wolica Nature Reserve is located in central Poland, within the Chojnów Forest District. It protects remnants of a natural deciduous woodland typical of the Mazovian region. The dominant tree species include pedunculate oak (*Quercus robur* L.) and Scots pine (*Pinus sylvestris* L.), but the presence of European ash (*F. excelsior*), which occurs here as mature, well-developed specimens, is particularly notable. Ash is an important component of the *Tilio-Carpinetum* forest community, and its presence in the reserve holds significant ecological and monitoring value, especially in the context of widespread ash dieback across Europe. The understorey features hornbeam (*Carpinus betulus*), small-leaved linden (*Tilia cordata*), and hazel (*Corylus avellana*), while the herb layer is rich in indicator species of fertile mixed forests.

Soil conditions in the study stands vary from fertile, well-drained loamy soils typical for the Białowieża Primeval Forest to more heterogeneous substrates in the Wolica Nature Reserve, reflecting the mosaic nature of central Polish forest soils. These differences in soil type and forest stand composition provide a valuable environmental gradient for assessing the impact of biotic and abiotic factors on ash vitality and genetic diversity.

To broaden the scope of genetic differentiation analyses, two additional reference ash populations were included: one from Kozienice FD (central Poland) and one from Czerwony Dwór FD (eastern Poland) (Table 1). These reference populations were particularly relevant for genetic differentiation analyses under Hardy–Weinberg equilibrium assumptions, enabling the assessment of potential genetic distinctiveness of BPF populations, especially in relation to vitality degree differentiation as defined by Roloff [5]. Inclusion of these populations from different forest districts provides a comparative framework to evaluate whether fragmentation and local environmental conditions in the BPF area influence genetic structure, and how these factors correlate with observed patterns of ash dieback and tree vitality. This multi-site approach strengthens the robustness of conclusions regarding genetic resilience and aids in identifying conservation priorities within varying landscape contexts.

Field vetting was conducted to determine the actual number of living ash trees. In each selected area of the observation plot, trees were permanently marked with paint certified for use in forests and the geographical coordinates of each tree were recorded using a GPS receiver. Live ash trees belonging to biosocial group I or II (according to Kraft’s classification) were designated in all investigated stands from BPF and Nature Reserve (Table 1). From all sampled mature trees (>20 cm DBH), wood samples (including the phloem) were taken with a Pressler incremental auger by taking a core sample from the trunk at breast height on the south side. All collected wood samples were transferred to the laboratory and stored at −80 °C before further analysis.

### 2.2. Health Status Classification of Ash Trees

**Figure 2:**
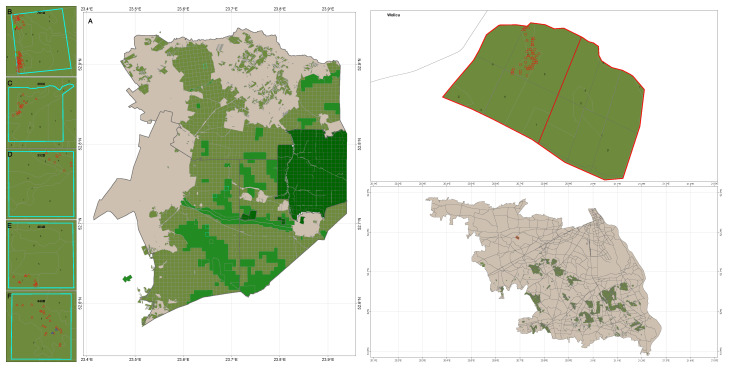
Detailed localization of European ash populations, assessed both by genetic analysis and vitality degree evaluation. (**A**) Białowieża Primeval Forest: Browsk FD (compartment 761A) and Hajnówka FD (compartments 306C, 332D, 464D, and 440D). (**B**–**F**) Different compartments of BPF. On the right: Nature Reserve of Wolica (Chojnów FD).

The health status of 61 and 65 ash trees (*F. excelsior*) from Browsk and Hajnówka Forest Districts, respectively (Figure 2), and 60 ash trees from the Wolica Reserve [18], was assessed using a method similar to that applied in the Mircze Forest District [26]. To further enhance the assessment, we incorporated concepts from Roloff’s framework for tree vitality and crown architecture [27,28], which considers phased vitality stages and crown structural changes as indicators of tree health.

Regarding vitality, ash trees are divided into the following grades:

Degree 0—vital tree;
Degree 1—weakened tree;
Degree 2—damaged tree;
Degree 3—dying or dead tree.

### 2.3. Assessment of Genetic Variation Based on Allelic Diversity

Total DNA from each wood sample was extracted using NucleoSpin Plant II (Macherey-Nagel, Düren, Germany), following the manufacturer’s protocol with some modifications: 600 μL PL2, 150 μL PL3, and 900 μL PC buffer. Nuclear and chloroplast microsatellite loci were amplified in two multiplexes. The first multiplex consisted of five loci—Femsatl-4, Femsatl-8, Femsatl-19, ccmp3, ccmp6; and the second one consisted of another five loci—Femsatl-11, Femsatl-16, M2-30, ccmp7, and ccmp10 [29]. PCR conditions and genotyping procedures were consistent with Pacia et al. [18]. All PCR reactions were performed in Veriti 96 Thermal Cycler (ThermoScientific, Waltham, MA, USA) and genotyping in CEQ™8000 (Beckman Coulter®, Fullerton, CA, USA) sequencer.

Genetic diversity within populations was quantified using a suite of standard population genetic parameters derived from allelic variation. Specifically, the observed number of alleles per locus (*N*_a_), the effective number of alleles (*N*_e_), the mean number of private alleles per locus (*A*_priv_), observed heterozygosity (*H*_O_), expected heterozygosity under Hardy–Weinberg equilibrium (*H*_E_), and Shannon’s information index (*I*) were calculated using GenAlEx v. 6.503 software package [30]. To account for potential biases introduced by unequal sample sizes in the estimation of population differentiation (*F*_ST_), allelic richness (*A*_R_) was standardized to a sample size of 16 individuals per population using the rarefaction method implemented in FSTAT v2.9.4 [31]. This software was also used to compute Nei’s gene diversity (G_d_) [32], and the inbreeding coefficient within populations (*F*_IS_) [33]. The statistical significance of *F*_IS_ values was evaluated using Hardy–Weinberg equilibrium tests based on 10,000 allele permutations per population. Confidence intervals for significance were set at 95% and 99%, allowing for robust inference of deviations from random mating within populations.

### 2.4. Genetic Differentiation Among Ash Populations

Genetic differentiation among populations was quantified using the fixation index (*F*_ST_), estimated via two complementary approaches: uncorrected and corrected for the presence of null alleles. Uncorrected *F*_ST_ values were computed using FSTAT v. 2.9.4 [31], while null allele-corrected estimates were obtained using FreeNA software, https://www1.montpellier.inra.fr/CBGP/software/FreeNA/ (accessed on 4 September 2025) [34]. The presence and frequency of null alleles were assessed in FreeNA through 100,000 bootstrap replicates. Subsequently, global *F*_ST_ values were adjusted using the Expectation-Maximization (EM) algorithm [35], following the method of Weir and Cockerham [33]. To further partition genetic variation within and among populations, an analysis of molecular variance (AMOVA) was conducted in GenAlEx v6.502 [36]. This analysis was based on pairwise genetic distances and employed 999 permutations to test the significance of variance components, with a statistical significance threshold set at *p* = 0.05.

For chloroplast loci, a set of additional parameters, i.e., π—nucleotide diversity, Φ_ST_ and *N*_ST_ as measures of haplotype similarity between vitality degrees based on haplotype frequency or mutations (respectively) were estimated in Arlequin ver. 3.5.2.2 [37].

### 2.5. Clustering of Individual Trees by Vitality Degree

Markov Chain Monte Carlo (MCMC) analysis has been completed using K-Means clustering to simulate population structure for nSSR multilocus genotype data across the vitality degree distribution. This was implemented in STRUCTURE v.2.3.4 [38] under the assumption of Hardy–Weinberg equilibrium [39], with a burn-in period of 10,000 iterations followed by 100,000 MCMC iterations. A range of cluster numbers (K = 1 to 8) was tested, with 5 replicates. The probability distributions of the data LnPr(Data) and the ΔK values were examined in the Structure Harvester Web v. 0.6.94 application [40,41].

To determine the major patterns within a multivariate data set for cpDNA markers, a Principal Coordinate Analysis (PCoA) was conducted in *R* version 2.8.0 (factoextra and NbClust packages, and vegan package [42]), where the optimal K-means clustering was determined using Silhouette version 6.503 software [43,44,45]. The R software was accessed via https://www.r-project.org/ (accessed on 4 September 2025).

For both marker types, Pearson’s correlation coefficient (r) and Tukey HSD tests were calculated to assess the relationships among genetic diversity parameters and their distribution across three vitality degrees (1, 2, and 3). All statistical analyses were performed using Python 3.12.3 with Scipy.stats [46] with *p* < 0.05.

## 3. Results

### 3.1. Health Status Classification of Ash Trees Based on Field Assessments

Vitality monitoring across Browsk, Hajnówka, and Chojnów (186 trees) revealed a balanced distribution of examined ashes among the three vitality degrees, with dying trees (3rd degree) slightly predominant (36%) over the others. Browsk and Chojnów had higher proportions of weakened trees (degree 1), while Hajnówka had more than half of dying individuals (Table 2).

### 3.2. Genetic Diversity Parameters in Five Populations Assessed with Nuclear and Chloroplast SSR Markers

Analysis of six nSSR loci across five populations (Browsk, Hajnówka, Chojnów, Kozienice, Czerwony Dwór) revealed moderate to high diversity, with 100% locus polymorphisms in 185 successfully amplified genotypes. Hajnówka exhibited the highest allelic richness (*A*_R(16)_ = 20.571) and the highest private alleles number (*A*_priv_ = 3.000), which aligns with the high effective allele number (*N*_e_ = 10.309) for this population (Table 3). Czerwony Dwór, with the smallest sample (N = 16), showed reduced richness and private alleles. All analyzed populations shared a high proportion of private alleles (*A*_priv_ = 2.100) in nSSR loci. The distribution of *A*_priv_ across the five *F. excelsior* stands revealed notable differences in genetic uniqueness, showing the highest number of private alleles (18 alleles across multiple nSSR loci, *A*_priv_ = 3.000) in Hajnówka (Table 3 and Table A1), suggesting a genetically distinct gene pool in this stand. This aligns with high allelic richness (*A*_R(16)_ = 20.571) and effective allele number (*N*_e_ = 10.309) found in this population (Table 3). The population of Kozienice also exhibited a rich set of private alleles (16 alleles), particularly concentrated in the Femsatl-F4 locus, where one allele (191 base pairs) reached a frequency of 0.204, probably as an effect of selection or allelic fixation (Table A1). By contrast, Chojnów and Browsk populations had moderate numbers of private alleles (13 and 7, respectively), with lower frequencies, suggesting ongoing gene flow or shared ancestry with neighboring populations. Czerwony Dwór, despite its small sample size, presented 9 private alleles, some with relatively high frequencies (e.g., 185 bp at 0.094), indicating local differentiation despite reduced overall diversity (Table A1). Observed heterozygosity (*H*_O_ from 0.609 to 0.713) was consistently lower than expected (*H*_E_), yielding a significant total inbreeding coefficient (mean *F*_IS_ = 0.178, *p* < 0.001) (Table 3).

Across 281 genotyped individuals, a percentage of 8.4 null alleles was detected and corrected for final *F*_ST_ value estimation. Then, global AMOVA results confirmed that most genetic variation (94%) resides within populations, with 6% variation among populations (*p* = 0.001), consistent with low population differentiation (*F*_ST_ = 0.044) found among all populations (Table A2).

Chloroplast markers shared a lower 0.89% of null alleles and higher population differentiation than nuclear loci among 186 determined haplotypes. The haplotype (*H*_D_) and nucleotide (π) diversity were highest in Kozienice FD (0.633 and 2.594, respectively) and in Chojnów FD (0.533 and 2.301), where nearly every individual had a unique haplotype, suggesting strong maternal lineage diversity (Table 4). Czerwony Dwór exhibited the lowest diversity *H*_D_ = 0.164 and π = 0.693, likely due to a limited number of haplotypes (*N*_a_ = 2.000).

Population of ashes from Kozienice stood out with eight private cpDNA alleles, many at high frequencies (e.g., ccmp3—94 bp and ccmp7—116 bp at 0.434), suggesting strong maternal lineage differentiation and possibly limited seed dispersal from this region (Table A3). Hajnówka had four private alleles, each at low frequency, indicating moderate haplotype uniqueness. Browsk, Chojnów, and Czerwony Dwór had only one private allele each, all at low frequencies, reflecting shared maternal lineages or recent colonization events.

The cpDNA data reinforce the phylogeographic structure observed in the AMOVA results (2% of variation, Table A4), in that all populations were moderately differentiated (*H*_D_ = 0.425, *p* = 0.060) and Φ_ST_ = 0.228 (*p* < 0.0001). The *N*_ST_ parameter (which takes into account the mutational differences between haplotypes) indicated a mean level (0.321) of genetic differentiation among the studied populations.

In the population genetics of *F. excelsior* across Europe, the assignment of chloroplast DNA (cpDNA) haplotypes to postglacial refugia was based on geographic distribution patterns, haplotype diversity, and historical migration routes inferred from phylogeographic studies identified four major refugia as Iberian Peninsula, Italy (Apennine), Balkans, and eastern Alps (potentially Dinaric Alps for some) [47,48]. Rare haplotypes (H06–H12) are not explicitly assigned but occur near eastern refugia, implying Balkan or eastern Alpine origins. Novel variants from the dataset (H13–H28), not reported in the literature, were deduced based on their similarity to the distribution after [29,49,50]: those in eastern/northern populations (Browsk, Hajnówka, Czerwony Dwór) were assigned to the Balkans, while central (Kozienice, Chojnów) ones aligned with Italian (Apennine) patterns (Table 5).

### 3.3. Comparison of Genetic Diversity Parameters Among the Degrees of Tree Vitality

Mean values of the genetic parameters studied in three populations of ashes (Browsk, Hajnówka, and Chojnów) classified in three degrees of vitality (Table 1), and assessed by nuclear and chloroplast DNA markers, were collectively presented in Table 6. Genetic nSSR diversity parameters were consistently high across all vitality degrees, with slight increases in effective allele number (*N*_a_) and Shannon’s index (*I*) from vitality degree 1 to 3. The expected heterozygosity and gene diversity reached high values (mean *H*_E_ = 0.835 and mean *G*_d_ = 0.844, respectively), and the global *F*_IS_ was 0.103 (*p* < 0.001), revealing a statistically significant excess of homozygotes.

The analysis of private alleles across three vitality classes after Roloff [5] revealed subtle but informative patterns, i.e., the highest number of private alleles (17, *A*_priv_ = 2.833) was found in vitality degree 2 (damaged trees), suggesting that genetic nSSR diversity is not linearly correlated with vitality (Table 6 and Table A5). This group of trees may represent a transitional genetic pool, maintaining both common and rare alleles. Vitality degree 3 (dying trees) also showed a rich set of private alleles (15), including several of them in loci Femsatl-F4, Femsatl-F11, Femsatl-F19, and M2-30 (Table A5). This could reflect genetic drift, inbreeding, or local adaptation under stress. Vitality degree 1 (weakened trees) had 10 private alleles, indicating moderate genetic distinctiveness, possibly maintained by stabilizing selection or higher gene flow (Table A5).

However, genetic differentiation among vitality groups assessed by nSSR markers was very low (*F*_ST_ = 0.009, *p* = 0.05), supported by the AMOVA test (1%, Table A6), suggesting that tree vitality was not strongly associated with nuclear genetic variation (Table 6).

Chloroplast DNA metrics showed elevated haplotype richness in class 3 (*N*_a_ = 5.250, *A*_priv_ = 1.250), but low differentiation (Φ_ST_ = 0.003, *p* = 0.196), indicating random haplotype distribution across vitality classes (Table 6). However, haplotype diversity (*H*_D_ = 0.569) was relatively high, in concordance with high nucleotide diversity π = 0.569 across all vitality degrees. The obtained *N*_ST_ < Φ_ST_ pattern (Table 6) also confirmed that haplotypes are not clustered by vitality degrees, and their distribution is likely random across vitality groups. This implies that maternal lineage variation does not correspond to tree health status, and vitality classification does not reflect underlying cpDNA divergence. Only 10 private chloroplast alleles were denoted among three vitality degrees, with the highest share of 5 haplotypes *A*_priv_ = 1.250 in the 3rd degree (Table 6 and Table A7), indicating an accumulation of evolutionarily important haplotypes from the adaptation point of view in the lowest health group of trees.

Only one of the above parameters obtained for nSSR and cpDNA markers showed statistical significance (Table 7). For nSSR markers, allelic parameters exhibited a modest increase with declining vitality (e.g., *N*_a_ from 20.667 in Degree 1 to 22.167 in Degree 3; Table 6), was accompanied by a significant positive Pearson correlation for Shannon’s index (r = 0.9981, *p* = 0.0397), indicative of rare allele accumulation via genetic drift in stressed individuals [5]. CpDNA patterns showed non-significant correlations (all *p* > 0.05), but negative trends in haplotype diversity (*H*_D_, r = −0.8864) and nucleotide diversity (π, r = −0.8660) imply progressive maternal lineage depletion in Degree 3 (*H*_D_ = 0.547 vs. 0.610 in Degree 1 from Table 6).

**Figure 3:**
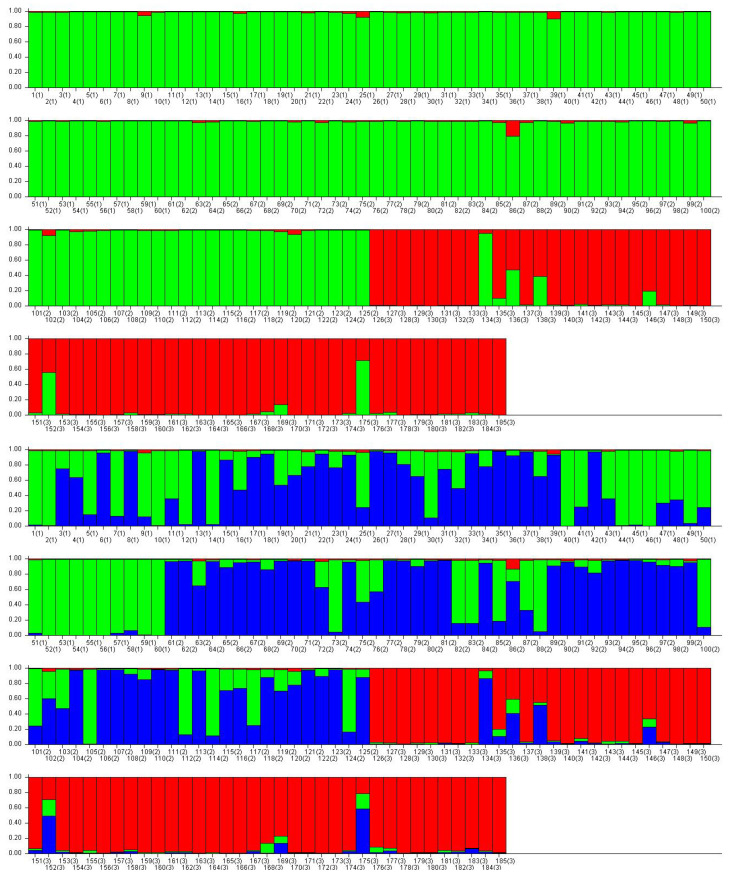
Bayesian clustering of 185 *F. excelsior* individuals using STRUCTURE v. 2.3.4 based on nuclear microsatellite (nSSR) markers, referring to the degrees of vitality (1—green, 2—blue, and 3—red bars); with K = 2, and K = 3 inferred clusters. The primary number on the X axis denotes individual trees sampled from population Browsk FD, Hajnówka FD, and Chojnów FD. The value in parentheses, e.g., (1), (2), (3), indicates the vitality degree of each tree.

**Figure A1:**
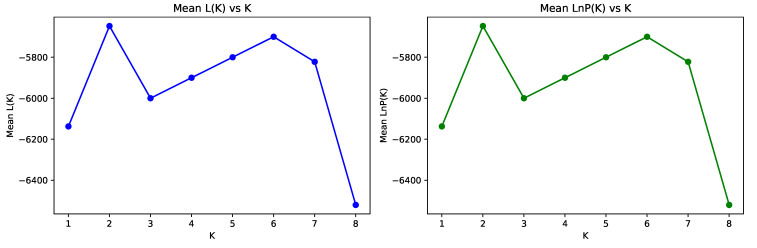

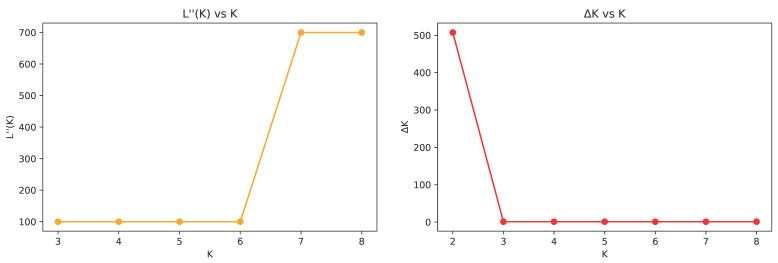
Comparative plots for K = 1 to 8 assignments after Evanno et al. [40] to estimate K number of clusters best fitting the nSSR STRUCTURE-based data: mean of estimated LnP(K) probability; mean likelihood values for each K; similarity coefficient; and ΔK statistics, as indicated above subfigures.

To this end, we conducted a more detailed analysis of tree distribution across the three vitality degrees using Bayesian clustering on nSSR genotype data from 185 individuals across three vitality degrees: 1 (weakened trees, N = 58), 2 (damaged, N = 61), and 3 (dying, N = 66), sourced from populations Browsk, Hajnówka, and Chojnów (Figure 3). The strongest structure clustering observed for K = 2 (Figure A1), which effectively differentiates the vitality degrees, with degree 3 (dying trees) largely assigned to one cluster, while classes 1 and 2 share the other, reflecting potential genetic erosion in dying trees (Figure 3).

However, K = 3 was more appropriate to explain a partial segregation of clusters (Figure 3). In Degree 1 (weakened trees, mainly in the top panel, including 60 individuals), a dominant green cluster (∼60–80% ancestry in most bars) suggests relatively homogeneous genetic backgrounds, potentially linked to higher resilience through maintained heterozygosity (*H*_E_ > 0.820, as in Table 6). Interspersed blue and minor red segments imply ongoing introgression, possibly from neighboring stands, supporting low differentiation (*F*_ST_ = 0.005) and resistance to pathogen-induced bottlenecks.

For Degree 2 (damaged trees, second panel, 66 individuals), the pattern shifts toward increased blue cluster dominance (∼50–70%), with green and red admixtures creating a mosaic of genotypes (Figure 3). This intermediate admixture may reflect transitional genetic pools under moderate stress, where private alleles (*A*_priv_ = 2.833) accumulate due to drift or Wahlund effects, aligning with elevated inbreeding (*F*_IS_ = 0.203, *p* < 0.001).

In Degree 3 (dying trees, third and fourth panels, 60 individuals), red cluster prevalence (∼70–90% in many trees) indicates stronger genetic structuring, with reduced admixture suggesting fixation of deleterious alleles or pathogen-driven selection purging diversity. Scattered green/blue intrusions highlight remnant gene flow, potentially accelerating decline via inbreeding depression (*F*_IS_ = 0.164, *p* < 0.001). Interestingly, in this Vitality Degree bar plots some dying trees, e.g., from Browsk (bar 134(3), corresponding to tree no. 59 from the stand); Hajnówka (bar 136(3), tree no. 9, bar 138(3), tree no. 14, bar 152(3), tree no. 45), and Chojnów (bar 175(3), tree no. 5) had higher ancestry proportions (>70%) in a unique cluster (blue) compared to weakened (1st degree) and damaged trees (2nd degree) with <50% per cluster. These particular trees exhibited distinct genotypes, including private alleles at Femsatl-F4, Femsatl-16, and Femsatl-19.

**Figure 4:**
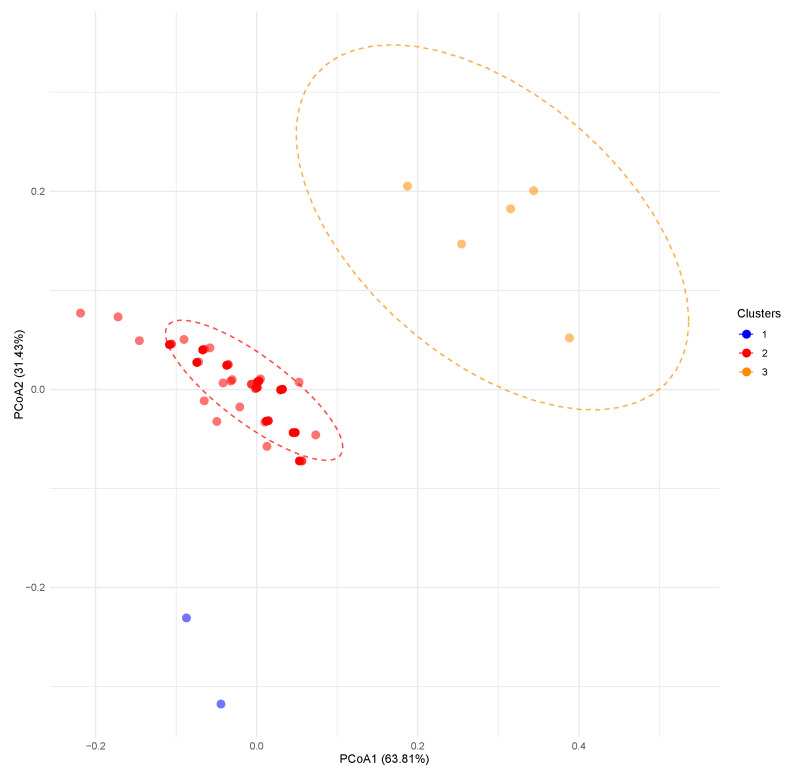
The Principal Coordinate Analysis (PCoA) plot based on cpDNA markers delineating the genetic distances among studied *F. excelsior* individuals categorized into three vitality classes [5]: weakened (Cluster 1), damaged (Cluster 2), and dying (Cluster 3).

PCoA plot derived from chloroplast DNA markers, reflects the previous clustering, where the PCoA1 accounts for 63.81% of the total variance, capturing the primary axis of haplotype differentiation, while PCoA2 explains 31.43%, together representing over 95% of the cumulative variation (Figure 4). Points are dispersed with moderate overlap between clusters, particularly between Clusters 1 and 2 along the negative to zero range of PCoA1 (−0.2 to 0.0), indicating shared maternal lineages and limited haplotype divergence in healthier vitality classes. Cluster 3 shows a slight shift toward positive PCoA1 values (0.0 to 0.4), suggesting subtle structuring potentially driven by fixation of specific haplotypes under stress, such as those associated with reduced nucleotide diversity (π = 2.264 in Degree 3, Table 6). Overall, the absence of discrete clustering aligns with non-significant differentiation metrics (Φ_ST_ = 0.003 ns, *N*_ST_ = 0.014; Table 6), implying random haplotype distribution across vitality degrees and minimal maternal lineage partitioning by health status.

## 4. Discussion

### 4.1. Adaptive Response Limitations and Pathogen Impact in Białowieża Ash Stands

Given that adaptive responses primarily occur in natural populations, monitoring tree health in forest reserves remains a critical scientific priority. In Poland and Belarus, ash-dominated stands within protected areas such as the Białowieża Primeval Forest (BPF) are increasingly threatened by compounded pressures, including habitat fragmentation and climate change.

*Hymenoscyphus fraxineus* was likely introduced unintentionally from Asia into Western Europe [52]. In Belarus, mass dieback of ash (*F. excelsior*) was first recorded in 2003 [53], with mortality in the Belarusian section of BPF rising sharply from 6.8% in 2003 to 12.2% in 2004—substantially exceeding the baseline annual rate of 1.3%. Over 80% of surveyed stands were classified as resilience-impaired, with only 12% considered biologically viable [54]. Damage to mature trees was severe: 90% exhibited branch necrosis averaging 42.9 ± 4.2%, predominantly linked to *H. fraxineus*. By 2005, forecasts pointed to the near-complete disappearance of ash stands within two to three years [10].

According to forest management records (Forest Management Project, 2015), the condition of ash stands across the BPF is now considered critical. Over the last survey period, the loss of ash on 605 hectares led to its replacement by secondary species, primarily black alder (*Alnus glutinosa*). Of the remaining stands, 21.2% were classified as having impaired structural stability. Most surviving ash populations showed signs of root rot and infestation by stem pests, contributing to the decline of ash as a dominant forest formation and its replacement by ecologically distinct assemblages.

This shift is also reflected in the accelerated transformation of ash–spruce communities into maple–hornbeam (*Acer-Tilia-Carpinus*) formations. Regeneration of ash is further restricted by intensive browsing from hoofed mammals, suppressing undergrowth and preventing the establishment of future cohorts [55].

In 2010, molecular genetic analyses confirmed the widespread presence of *H. fraxineus* throughout Belarus [56]. These studies revealed considerable intraspecific genetic diversity within the pathogen population, with RAPD analyses showing inter-strain variation between 7% and 47% of loci (D_N_ = 0.0715–0.4769). This diversity, together with the geographic clustering of genotypes, supports the hypothesis of multiple independent introductions of *H. fraxineus* isolates [11].

By contrast, the Polish section of BPF lacks comparable longitudinal records of ash dieback, underscoring the need for urgent genetic assessment. Population-level studies could illuminate adaptive mechanisms to biotic stressors, as invasive pathogens are known to exacerbate genetic erosion in fragmented forest habitats [12].

Health assessments in Browsk and Hajnówka (representing Białowieża Primeval Forest, BPF) versus Chojnów (potentially analogous to Wolica Nature Reserve, given its protected status) revealed nuanced differences in vitality distribution. In BPF stands, weakened trees (degree 1) comprised 31% overall, but Hajnówka showed a predominance of dying individuals (55%), likely exacerbated by dense canopy and pathogen accumulation from *H. fraxineus*. Browsk had higher proportions of weakened (44%) and damaged (31%) trees, suggesting better resilience, possibly due to microhabitat variation. In contrast, Chojnów exhibited balanced vitality (38% weakened, 35% damaged, 27% dying), with fewer dying trees than Hajnówka, aligning with lower dieback severity in managed reserves. This pattern echoes European trends, where protected forests like BPF experience higher mortality (up to 40% dying) compared to fragmented reserves (20–30%), attributed to reduced gene flow in old-growth stands [57,58]. Differences may stem from edaphic factors or pathogen inoculum levels, as observed in Danish ash, where vitality correlated with soil moisture [59]. In fact, many different environmental factors, including those favoring *H. fraxineus* life-cycle in forest ecosystem are crucial for ash dieback phenomenon [60,61]. In the case of BPF’s higher dying fraction (36% total) versus Chojnów’s ash trees implies greater vulnerability in ancient forests, necessitating targeted conservation, based on genetic data. An important complementary approach to field observations of ash dieback symptoms is artificial inoculation of *F. excelsior* genotypes with the causative pathogen, *H. fraxineus*. This method allows for controlled assessment of genotype resistance by simulating pathogen exposure under standardized conditions, which can help differentiate inherent genetic tolerance from environmental influences [62,63]. However, while artificial infection provides valuable insights into resistance mechanisms, it may not fully replicate the complexity of natural infection dynamics and interaction with other biotic and abiotic stressors [64]. Therefore, combining artificial inoculation with long-term field monitoring, as performed in this study, offers a comprehensive framework for understanding genetic resilience and informing conservation strategies [15,62]. Dendrochronological analyses have proven effective in reconstructing the history of ash dieback impacts by assessing growth reductions and anatomical changes in wood structure [65]. Moreover, this method helps interpret the effects of environmental factors on tree health and disease progression in near-natural forest remnants [28]. The application of dendrochronology thus provides critical temporal insights complementing field and genetic data in forest disease studies.

### 4.2. Genetic Diversity Levels in Studied Ash Populations Compared to Other Studies

Genetic diversity parameters assessed with six nuclear simple sequence repeat (nSSR) and four chloroplast DNA markers in the studied five *F. excelsior* populations revealed moderate to high levels of genetic diversity. The genetic diversity in Browsk and Hajnówka FD (both from BPF), in Chojnów FD (from Wolica Nature Reserve) and in Kozienice and Czerwony Dwór (two reference populations) aligns with European trends, where high heterozygosity may buffer dieback susceptibility [19]. All populations assessed by nSSR markers have shown substantial polymorphism across loci with mean expected heterozygosity (*H*_E_ = 0.826) and allelic richness (*A*_R(16)_ = 16.382). These values align with previous investigations on European ash, where *H*_E_ ranged from 0.700 to 0.850 in fragmented populations across Europe, reflecting resilience despite habitat loss and suggesting that outcrossing tend to maintain diversity even under progressing pathogen pressure [66,67]. All studied populations potentially reflected inbreeding depression or Wahlund effect, proven by a significant excess of homozygotes in all populations (mean *F*_IS_ = 0.178, *p* < 0.001), largely higher than observed in previous studies [21,47]. Especially, Hajnówka’s elevated allelic richness (*A*_R(16)_ = 20.571) and the highest private allele number (*A*_priv_ = 3.000) suggest a genetic uniqueness of this population, possibly due to historical isolation and/or an adaptation to the local environmental conditions [68,69]. Moreover, the significant inbreeding (*F*_IS_ > 0) in all ash populations suggests fragmentation impacts, potentially increasing their vulnerability (e.g., in Hajnówka, half of the trees were dying). Low nuclear differentiation between all five ash populations (*F*_ST_ = 0.044) underscores pollen dispersal efficacy [70], contrasting with higher cpDNA Φ_ST_ (0.228), indicative of seed-limited migration and post-glacial legacy [71]. This situation was commonly observed in many European ash populations [29,72,73,74], and in other deciduous trees, like white oak [75,76] or European beech [77,78,79].

Interestingly, in Browsk, Hajnówka and Chojnów, the cpDNA haplotypes H01–H05 showed clear geographic patterns, as they are prevalent in Italy and western Europe, linked to Apennine or Balkan refugia; with H02 and H05 clustering in central Europe, suggesting eastern Alpine sources; while H01 dominates eastern and northern Europe, pointing to Balkan Peninsula [47]. The novel haplotypes were present in all populations except Chojnów, which is situated along potential recolonization corridors from the south-central refugia. The higher haplotype diversity (*H*_D_ = 0.633) and private alleles (*A*_priv_ = 2.000) in Kozienice suggest a refugial signature consistent with Italian origins, where a possible post-recolonization bottleneck preserved unique variants of alleles. This spatial structuring mirrors the postglacial expansion of ash, where Balkan and Apennine lineages contributed to central European gene pools, facilitated by seed dispersal across the Alps and Carpathians.

Generally, the cpDNA haplotypes showed lower variability (mean *H*_D_ = 0.425, π = 1.793) than in the case of nSSR markers, consistently with maternal inheritance and limited seed dispersal in European ash. Kozienice and Chojnów exhibited the highest *H*_D_ (0.633 and 0.533, respectively) among other stands, mirroring phylogeographic patterns observed in French ash populations (*H*_D_ = 0.50–0.70), where glacial refugia influenced haplotype richness [49]. In contrast, low *H*_D_ (0.164) in Czerwony Dwór was substantially due to low tree number, and resembled bottleneck in European beech (*Fagus sylvatica*) stands in Greece (*H*_D_ = 0.20–0.40) [80]. Chloroplast DNA differentiation (Φ_ST_ = 0.228) among studied stands was higher than in nSSR (*F*_ST_ = 0.044), aligning with the level of differentiation from 0.225 to 0.888 for *F. excelsior* [21,49], and *F*_CT_ = 0.750 *Quercus robur* [81], highlighting strong structuring in maternal lineages. In this scope, Polish ash retains adaptive potential comparable to pan-European conspecifics, despite ongoing fragmentation and ADB pressure.

Taking into consideration the health-assessed populations, Browsk, Hajnówka, and Chojnów, demonstrated significantly higher allelic richness (*A*_R(16)_), with a mean of 18.641 compared to 12.994 in reference populations Kozienice and Czerwony Dwór (t = 4.262, *p* = 0.024) for the nSSR markers. Other nSSR metrics, such as mean number of alleles (*N*_a_ = 20.500 vs. 14.833, *p* = 0.138), effective alleles (*N*_e_ = 8.770 vs. 7.256, *p* = 0.320), private alleles (*A*_priv_ = 2.111 vs. 2.084, *p* = 0.975), and expected heterozygosity (*H*_E_ = 0.825 vs. 0.827, *p* = 0.938), showed no significant differences, although trends favored higher allelic counts in the health-assessed group. In contrast, cpDNA parameters revealed no significant distinctions among all analyzed populations. Haplotype diversity (*H*_D_) averaged 0.442 in health-assessed populations versus 0.399 in references (t = 0.235, *p* = 0.830), with Kozienice displaying the highest value (*H*_D_ = 0.633). Similarly, *N*_a_ (3.917 vs. 3.750, *p* = 0.908), *N*_e_ (1.672 vs. 1.988, *p* = 0.217), and *A*_priv_ (0.500 vs. 1.000, *p* = 0.582) were comparable, underscoring random maternal lineage distribution without vitality-linked structuring.

Generally, populations Browsk, Hajnówka, and Chojnów, examined under the vitality degrees, had comparable genetic diversity level in nSSR and in cpDNA markers as two reference populations, except Czerwony Dwór’s lowest parameters, like *N*_a_ = 11.833; *I* = 2.085 for nSSR markers, or *N*_a_ = 2.000 and *I* = 0.085 for cpDNA loci, which was due to the low sample size (N = 16 and 18, respectively) in this population.

These observed patterns imply that the health-assessed populations, particularly Hajnówka, are distinguished by elevated nuclear allelic richness, which may confer adaptive potential amid ash dieback pressures. This aligns with previous observations in Chojnów FD [18], or in 30 other Polish populations examined by Meger et al. [82]. The same trend was reported in Bavarian European ash studies, where nSSR allelic richness and observed heterozygosity were higher in less susceptible stands than in populations more affected by dieback [16]. In Bulgarian stands, microsatellite differentiation accounted for 8.7% of total variation, but specific allelic richness values were not detailed, suggesting moderate structuring similar to the low *F*_ST_ (0.044) here. Hyrcanian rear-edge populations harbored high diversity (e.g., comparable *H*_E_ > 0.80), emphasizing refugial roles, though direct AR comparisons are limited. Overall, the observed allelic richness highlights Browsk, Hajnówka, and Chojnów populations as reservoirs of nuclear diversity, warranting prioritization in conservation genetics for forest trees under environmental stress [3].

### 4.3. Genetic Variability and Differentiation for nSSR and cpDNA Across Vitality Degrees

Nuclear SSR diversity increased slightly with declining vitality, with dying trees (vitality degree 3) showing higher *N*_a_ (22.167) and *A*_priv_ (2.500) than weakened ones (*N*_a_ = 20.667, *A*_priv_ = 1.667; Table 5), potentially reflecting drift or selection purging maladaptive alleles. Bayesian clustering (at K = 3) revealed distinct cluster dominance in degree 3 (∼70–90% ancestry), indicating fixation of stress-associated genotypes, while degrees 1 and 2 exhibited admixture of ∼60–70% other genotypes. Comparable low differentiation among three vitality classes (*F*_ST_ = 0.009) reflects the structuring in Latvian *F. excelsior* (*F*_ST_ = 0.045) and Greek *F. angustifolia* (*F*_ST_ = 0.059) under dieback [83,84]. CpDNA showed random haplotype distribution (Φ_ST_ = 0.003 ns), with PCoA displaying overlap and slight shift in Vitality Degree 3 along PCoA1 (63.81%), suggesting that maternal lineages are not vitality-linked, unlike the previous study where cpDNA clustered by health class [18].

However, a positive correlation for Shannon’s index (r = 0.998, *p* < 0.05) implies rare allele accumulation in stressed trees, e.g., dying trees from Browsk, Hajnówka, and Chojnów which exhibit high admixture (>70%) of different genotypes, may be prone to superior survival against ash dieback, as they may harbor some resistance loci. Genome-wide SNP arrays or whole-genome resequencing would refine *F*_ST_ estimates, identifying outlier loci under selection in Vitality Degree 3 clusters, as demonstrated in UK ash genomes [19]. Contemporary NGS studies, including GWAS on ash genome, have localized resistance to chromosomes 1 and 7, associating SNPs with reduced susceptibility via defense genes like NBS-LRR [85]. Looking forward, these findings open avenues for advanced “omics” integration to dissect vitality-genetics interplay. Transcriptomics has already profiled leaf responses to *H. fraxineus*, identifying differential expression in tolerant genotypes during early infection stages, with upregulated pathways in several phenylpropanoid biosynthesis [86]. Metabolomics could extend this by quantifying secondary compounds (e.g., secoiridoids) correlating with *H*_E_ and vitality, as preliminary studies link iridoid glycosides to reduced lesion formation during *H. fraxineus* infection [19,87]. Proteomics might reveal post-translational modifications in stress proteins, while epigenomics could explore methylation patterns influencing *F*_IS_ elevation in damaged trees (Vitality Degree 2), potentially heritable across generations [13]. Multi-omics fusion, via machine learning integration, promises predictive models for vitality trajectories, enhancing breeding programs for dieback tolerance [85]. Such approaches, combined with microbiome analyses of endophytes modulating resistance (e.g., via mycobiome shifts), could inform holistic conservation strategies, bridging molecular insights with ecosystem-level resilience [88,89].

Based on the present knowledge, the future of *F. excelsior* in Europe is not optimistic in the short term (10–20 years), with further population declines of 70–90% due to high pathogen virulence and low natural resistance (below 5% of trees) [4]. The accumulation of many unfavorable biotic and abiotic stress factors in protected ash stands, like in BPF [90,91,92], combined with non-reduced virulence of *H. fraxineus* in Europe, despite its genetic bottleneck founded by just two haplotypes from Asia [93].

However, in the longer term (50+ years), genetic selection for tolerance, supported by breeding programs using transcriptomic markers (e.g., MADS-box genes regulating dormancy and senescence), may enable population recovery through hybridization with resistant Asian species [85]. In sites like the Białowieża Forest and Wolica Reserve, where biodiversity buffers effects, the ecological niche of ash may be partially preserved through regeneration from resistant individuals, but climate changes (droughts) will intensify mortality, requiring active conservation of the genetic pool [17,90,94]. Ultimately, without genomic intervention, the species may become rare, disrupting forest ecosystems, but with potential for evolutionary adaptation in isolated populations [15,95].

### 4.4. Health of Ash Trees in a Genetic Context

The health of European ash (*F. excelsior*) has become a major focus of forestry research in Europe, especially due to the widespread occurrence of ash dieback, a disease caused by the fungus *Hymenoscyphus fraxineus* (formerly *Chalara fraxinea*) [1].

Studies have shown that European ash populations are significantly more susceptible to infection than Asian species, which evolved in the presence of the pathogen and developed resistance mechanisms [17]. These genetic differences between populations suggest that genetic diversity plays a crucial role in tree resistance to disease.

European Forest Genetic Resources Programme (EUFORGEN) guidelines on the conservation of genetic resources of European ash emphasize the importance of maintaining genetic diversity in forest populations. The document highlights the species’ polygamous reproductive system, variability in sexual expression, and the need to select disease-resistant genotypes in breeding programs [96].

A report prepared by the University of Agriculture in Kraków for the Polish State Forests provides detailed biochemical and genetic analyses of ash dieback. It points to the varying pathogenicity of the fungus and the possibility of identifying naturally resistant genotypes [97].

All of the above gives hope that the trees that survived the ash dieback phenomenon may pass on tolerance or resistance to the disease to the next generation of ash trees in Europe. Recent genomic studies confirm that natural selection is already acting on ash populations, with resistant individuals showing heritable traits that could support long-term recovery [94,98]. In recent years, there has been a sharp increase in cases of ash (*F. excelsior*) dieback in southwestern Sweden, caused by the fungus *H. fraxineus*. Research conducted by Bengtsson et al. [57] showed that in 2020, as many as 94.5% of monitored trees exhibited symptoms of the disease, a significant rise compared to 62% in 2009. Over the course of a decade, 21% of the studied ashes had died, indicating a serious threat to the veteran tree population, especially those with high ecological and cultural value. In our case, it turned out that the trees undergoing dieback (Roloff damage level 3, known as the resignation phase) differ genetically. It is therefore likely that these ashes carry alleles that proved disadvantageous for survival in a rapidly changing environment—specifically, one altered by the introduction of a pathogen to Europe that ashes had never encountered before. As Stocks et al. [99] emphasizes, *H. fraxineus* originated in Asia, and European ash trees have not co-evolved with it, which likely explains their lack of effective immune responses.

We believe that, since the trees did not co-evolve with the pathogen, they never developed appropriate defense mechanisms. This is supported by genomic studies showing that resistance in ash is a polygenic trait, influenced by multiple loci [15,99]. It is likely that the surviving individuals differ precisely in their ability to cope with infections, which gives them the advantage of producing offspring and passing on tolerance-related genes to the next generation. Metheringham et al. [98] demonstrated that natural selection is already acting on ash populations, with younger trees showing higher genomic estimated breeding values (GEBVs) for resistance.

Furthermore, recent research by Franco Ortega et al. [100] identified specific gene families, such as MADS-box genes, and epigenetic markers that may play a role in tolerance mechanisms. These findings suggest that the surviving trees are not only genetically distinct but may also possess adaptive traits that enable them to withstand infection and contribute to the long-term resilience of the species.

Based on this concept, foresters have adopted the principle of preserving and protecting old ash trees as valuable veteran specimens that disperse seeds in the forest and enrich its biological diversity. Veteran trees are increasingly recognized not only for their ecological value but also for their potential role in maintaining genetic resilience within forest ecosystems [101].

However, we are aware that natural selection processes are long-term. Poland is in a favorable position in this regard, as ash dieback was first observed here over 30 years ago [1], making it more advanced in this respect than other European countries where the pathogenic fungus appeared later [90].

## 5. Conclusions

The studied *F. excelsior* populations exhibit robust genetic diversity, with nuclear markers indicating higher values of genetic parameters in Browsk, Hajnówka, and Chojnów populations from protected areas, and chloroplast data highlighting maternal structuring. Notably, Hajnówka exhibited the highest individual *A*_R(16)_ (20.571) and private allele count (*A*_priv_ = 3.000), suggesting localized allelic accumulation potentially driven by historical gene flow or local adaptation in BPF. Vitality degrees show minimal genetic differentiation, suggesting environmental factors dominate health decline. The most important results achieved in the study are summarized below:

- Despite the ongoing ash dieback, results indicate sustained moderate to high genetic diversity (*H*_E_ = 0.826), reflecting the adaptive potential of *F. excelsior* populations in Poland.
- Low inter-population differentiation (*F*_ST_ = 0.044 for nSSR; Φ_ST_ = 0.228 for cpDNA) suggests intensive gene flow, which may buffer against localized selection pressures induced by the pathogen.
- The absence of significant genetic differences between trees of varying vitality levels (nSSR *F*_ST_ = 0.009; cpDNA Φ_ST_ = 0.003) implies that resistance to dieback may be influenced by epigenetic or environmental factors rather than solely by genetic structure.
- The observed admixture of genotypes among dying individuals (STRUCTURE K = 3) may point to the presence of susceptible genetic lines, opening avenues for resistance-based selection.
- These findings support the implementation of selective breeding programs in forest nurseries, emphasizing the identification and propagation of genotypes resistant to *H. fraxineus*.

A deeper understanding of the genetic foundations of ash resistance has the potential to bridge the gap between epigenetics, genotype, and phenotype, thereby enhancing future genetic monitoring efforts aligned with conservation priorities. The integration of next-generation sequencing (NGS) and multi-omics approaches—such as transcriptomics, which can identify upregulated pathogenesis-related genes in tolerant ash genotypes, and proteomics, which can reveal protein biomarkers for early disease detection—offers promising avenues for targeted resistance breeding against ash dieback. These advanced genetic insights and omics-based strategies could support coordinated breeding programs across Europe, contributing to the long-term resilience of ash populations.

## Figures and Tables

**Table 1:** Geographical and environmental characteristics of all analyzed ash populations.

Population Feature	Forest District	Forestry	No. of Trees	Geo. Location	Mean Age ^2^ [Years]	Area [ha]
BPF ^1^	Browsk	Lewkowo	61	52°54′27″ N, 23°64′55″ E	74	2.44
BPF ^1^	Hajnówka	Nieznany Bór	65	52°44′1″ N, 23°40′36″ E	66	12.74
Nature Reserve	Chojnów	Wolica	60	52°11′15″ N, 20°41′18″ E	22	1.77
Reference	Kozienice	Przejazd	77	51°32′36″ N, 21°24′51″ E	93	10.30
Reference	Czerwony Dwór	Olszanka	18	55°7′21″ N, 22°12′11″ E	72	4.67

^1^ Białowieża Primeval Forest; ^2^ in 2018.

**Table 2:** Number of individuals assigned to vitality degrees based on ground surveys (2016).

Health Status (Degrees) ^1^	Population			Number of Trees
	Browsk	Hajnówka	Chojnów	
1—Weakened	27	8	23	58 (31%)
2—Damaged	19	21	21	61 (33%)
3—Dying	15	36	16	67 (36%)
Total	61	65	60	186

^1^ Classification of trees’ health status after Roloff [5].

**Table 3:** Genetic diversity parameters ^1^ for the studied *F. excelsior* populations assessed by nSSR markers. Population locations correspond to those listed in Table 1.

Population	N	*N*_a_	*N*_e_	*A*_R(16)_	*A*_priv_	*I*	*H*_O_	*H*_E_	*G*_d_	*F*_IS_
Browsk ^1^	55	19.333	9.060	18.197	1.167	2.370	0.713	0.838	0.847	0.151 ***
Hajnówka ^1^	62	23.167	10.309	20.571	3.000	2.453	0.689	0.847	0.855	0.191 ***
Chojnów	60	19.000	6.941	17.156	2.167	2.155	0.689	0.790	0.798	0.137 ***
Kozienice	58	17.833	7.406	12.681	2.667	2.260	0.609	0.835	0.845	0.231 ***
Czerwony Dwór	16	11.833	7.106	13.306	1.500	2.085	0.701	0.819	0.851	0.179 **
Mean	50	18.233	8.164	16.382	2.100	2.265	0.680	0.826	0.840	0.178 ***

^1^ Abbreviations: N—sample size (mean number of alleles); *N*_a_—number of different observed alleles; *N*_e_—number of effective alleles; *A*_R(16)_—allelic richness (standardized for 16 individuals); *A*_priv_—number of private alleles; *I*—Shannon’s diversity index; *H*_O_—observed heterozygosity; *H*_E_—expected heterozygosity; *G*_d_—gene diversity [32]; *F*_IS_—within-population Wright’s inbreeding coefficient (*** *p* < 0.001; ** *p* < 0.01 after Fisher’s method).

**Table 4:** Genetic diversity parameters ^1^ for the studied *F. excelsior* populations assessed by cpDNA markers. Population locations correspond to those listed in Table 1.

Population	N	*N*_a_	*N*_e_	*A*_priv_	*I*	*H*_D_	π
Browsk ^1^	60	3.750	1.803	0.250	0.680	0.372	1.593
Hajnówka^1^	64	4.250	1.958	1.000	0.756	0.421	1.786
Chojnów	60	3.750	1.255	0.250	0.897	0.533	2.301
Kozienice	76	5.500	2.750	2.000	1.146	0.633	2.594
Czerwony Dwór	18	2.000	1.225	0.000	0.293	0.164	0.693
Mean	55.6	3.850	1.998	0.700	0.754	0.425 **	1.793

^1^ Abbreviations: N—sample size (mean number of alleles); *N*_a_—number of different observed alleles; *N*_e_—number of effective alleles; *A*_priv_—number of private alleles; *I*—Shannon’s diversity index; *H*_D_—haplotype diversity [32], π—nucleotide diversity. ** *p* < 0.01.

**Table 5:** Chloroplast DNA haplotype classification ^1^ detected in the *F. excelsior* populations with their post-glacial refugium assignment.

Haplotype	ccmp3	ccmp6	ccmp7	ccmp10 ^2^	Frequency (%)	Populations	Phylogeographic Origin ^1^
H1	97	97	118	94	15.0	Browsk; Hajnówka; Chojnów; Kozienice	Balkans
H2	97	96	118	94	20.0	Browsk; Hajnówka; Chojnów; Kozienice	Balkans/Appenine
H3	97	97	117	93	7.6	Browsk; Hajnówka; Chojnów	Balkans/Appenine
H4	97	96	118	93	11.7	Browsk; Hajnówka; Chojnów	Balkans/Appenine
H5	97	97	118	95	11.0	Browsk; Hajnówka; Kozienice; Czerwony Dwór	Balkans/Appenine
H6	97	96	118	95	4.7	Browsk; Hajnówka	Balkans
H7	97	97	118	92	0.3	Browsk; Hajnówka	Balkans
H8	97	96	117	92	5.8	Browsk; Hajnówka	Balkans
H9	97	98	118	94	7.0	Browsk; Hajnówka	Balkans
H10	97	99	117	93	3.0	Browsk; Hajnówka	Balkans
H11	97	97	118	92	1.1	Browsk; Hajnówka	Balkans/Appenine
H12	96	97	118	95	2.0	Browsk; Hajnówka	Balkans
H13 ^3^	97	95	118	94	0.3	Browsk; Hajnówka	Balkans
H14	97	95	118	93	0.3	Browsk; Hajnówka	Balkans
H15	96	97	118	94	1.8	Hajnówka	Balkans
H16	96	97	118	93	1.1	Hajnówka	Balkans
H17	95	97	118	94	0.3	Hajnówka	Balkans
H18	102	97	118	94	0.7	Kozienice	Apennine
H19	102	97	118	95	0.3	Kozienice	Apennine
H20	102	101	118	95	0.3	Kozienice	Apennine
H21	96	99	118	94	0.3	Kozienice	Apennine
H22	97	96	123	99	0.3	Kozienice	Apennine
H23	96	97	118	95	0.3	Kozienice	Apennine
H24	102	99	118	93	0.3	Kozienice; Czerwony Dwór	Apennine
H25	96	98	118	93	0.3	Kozienice; Czerwony Dwór	Apennine
H26	97	100	118	94	4.0	Kozienice; Czerwony Dwór	Apennine
H27	97	100	118	93	0.3	Kozienice; Czerwony Dwór	Apennine
H28	97	100	117	94	0.3	Kozienice; Czerwony Dwór	Apennine

^1^ According to Heuertz et al. [47] and Tollefsrud et al. [50]. ^2^ A consistent +9 base pairs shift was applied to ccmp10 to align with literature patterns, reflecting potential genotyping calibration differences, e.g., in Sutherland et al. [29] and Gömöry et al. [51]. ^3^ Novel variants from H13 to H28, not reported in the literature.

**Table 6:** Genetic diversity parameters ^1^ for the *F. excelsior* populations assessed with nSSR and cpDNA markers in three different degrees of vitality [5].

Marker	Vitality Degree ^2^	*N*_a_	*N*_e_	*A*_priv_	*I*	*H*_E_	*G*_d_	*F*_ST_	*F*_IS_
nSSR	1	20.667	9.450	1.667	2.362	0.825	0.834	-	0.147 ***
	2	21.667	9.395	2.833	2.395	0.841	0.850	-	0.203 ***
	3	22.167	10.474	2.500	2.436	0.839	0.847	-	0.164 ***
	Mean	21.500	9.773	2.333	2.398	0.835	0.844	0.009 *	0.103 ***
cpDNA	**Vitality Degree**	***N*_a_**	***N*_e_**	***A*_priv_**	***I***	***H*_D_**	***π***	**Φ_ST_**	***N*_ST_**
	1	4.750	2.779	0.500	1.114	0.610	2.517	-	-
	2	4.000	2.433	0.250	1.008	0.550	2.264	-	-
	3	5.250	2.399	1.250	1.053	0.547	2.264	-	-
	Mean	4.677	2.537	0.667	1.058	0.569 ns	2.348	0.003 ns	0.014

^1^ Abbreviations: *N*_a_—number of different observed alleles; *N*_e_—number of effective alleles; *A*_priv_—number of private alleles; *I*—Shannon’s diversity index; *H*_E_—expected heterozygosity; *G*_d_—gene diversity of Nei [32]; *F*_IS_—within-population Wright’s inbreeding coefficient (after Fisher’s method); *H*_D_—haplotype diversity [23]; *π*—nucleotide diversity; *F*_ST_—genetic differentiation level for nSSR markers; Φ_ST_ and *N*_ST_—measure of haplotype similarity between degrees. *** *p* < 0.001; * *p* < 0.1; ns—not significant (*p* > 0.1). ^2^ classification after Roloff [5], 1—Weakened trees; 2—Damaged; 3—Dying.

**Table 7:** Pearson correlation coefficients for nSSR and cpDNA markers in linear associations between vitality degrees (1: weakened, 2: damaged, 3: dying).

Marker	Parameter ^1^	Pearson r	*p*-Value ^2^
nSSR	*N*_a_	0.9820	0.1210
	*N*_e_	0.8425	0.3622
	*A*_priv_	0.6935	0.5122
	*I*	0.9981	0.0397 *
	*H*_E_	0.8030	0.4065
	*G*_d_	0.7643	0.4462
	*F*_IS_	0.2961	0.8087
cpDNA	*N*_a_	0.3974	0.7399
	*N*_e_	−0.9036	0.2818
	*A*_priv_	0.7206	0.4878
	*I*	−0.5733	0.6113
	*H*_D_	−0.8864	0.3065
	π	−0.8660	0.3333

^1^ Parameters abbreviations like in Table 4 and Table 6. ^2^ Significance was evaluated at α = 0.05. * *p* < 0.1.

## Data Availability

The original contributions presented in the study are included in the article, further inquiries can be directed to the authors. The raw data supporting the conclusions of this article will be made available by the authors on request.

## Appendix A

**Table A1:** Summary of private alleles of nSSR loci distribution by population.

Population	Locus	Allele (pb)	Frequency
Browsk ^1^	Femsatl-F4	222	0.018
	Femsatl-F8	131	0.018
	Femsatl-F11	214	0.017
	Femsatl-F11	228	0.008
	Femsatl-F16	190	0.022
	Femsatl-F16	194	0.033
	M2-30	176	0.009
Hajnówka ^1^	Femsatl-F4	158	0.008
	Femsatl-F4	180	0.031
	Femsatl-F4	208	0.008
	Femsatl-F4	214	0.008
	Femsatl-F4	216	0.008
	Femsatl-F4	224	0.023
	Femsatl-F8	133	0.008
	Femsatl-F8	137	0.023
	Femsatl-F11	180	0.017
	Femsatl-F11	218	0.008
	Femsatl-F11	220	0.008
	Femsatl-F11	222	0.017
	Femsatl-F16	182	0.018
	Femsatl-F19	204	0.008
	Femsatl-F19	218	0.008
	M2-30	256	0.008
	M2-30	272	0.008
	M2-30	282	0.008
Chojnów ^2^	Femsatl-F4	234	0.008
	Femsatl-F8	147	0.009
	Femsatl-F8	168	0.009
	Femsatl-F8	197	0.121
	Femsatl-F11	224	0.017
	Femsatl-F16	176	0.008
	Femsatl-F19	158	0.008
	Femsatl-F19	187	0.025
	Femsatl-F19	196	0.033
	Femsatl-F19	202	0.008
	M2-30	192	0.017
	M2-30	194	0.017
	M2-30	260	0.008
Kozienice	Femsatl-F4	128	0.019
	Femsatl-F4	161	0.019
	Femsatl-F4	173	0.019
	Femsatl-F4	177	0.009
	Femsatl-F4	181	0.046
	Femsatl-F4	191	0.204
	Femsatl-F4	193	0.037
	Femsatl-F4	213	0.009
	Femsatl-F4	229	0.028
	Femsatl-F4	235	0.009
	Femsatl-F4	241	0.009
	Femsatl-F16	192	0.014
	Femsatl-F16	212	0.014
	Femsatl-F16	242	0.014
	Femsatl-F19	210	0.014
	M2-30	146	0.007
Czerwony Dwór	Femsatl-F4	175	0.031
	Femsatl-F4	185	0.094
	Femsatl-F4	187	0.031
	Femsatl-F4	189	0.063
	Femsatl-F4	205	0.031
	Femsatl-F4	221	0.031
	Femsatl-F4	223	0.031
	Femsatl-F4	225	0.031
	Femsatl-F11	230	0.033

^1^ Białowieża Primeval Forest; ^2^ Nature Reserve.

**Table A2:** Partitioning of genetic variation within and among the studied ash populations (AMOVA) assessed by nSSR markers.

Source of Variability (nSSR)	d.f. ^1^	Sum of Squares	Mean Square	Estimated Share (%)	Variation	*p* Value ^2^
Among populations	4	83.425	20.856	0.170	6%	
Within populations	547	1448.349	2.648	2.648	94%	
Total	551	1531.774		2.818	100%	0.001

^1^ d.f.—degrees of freedom. ^2^ *p* value based on 999 permutations.

**Table A3:** Summary of private alleles of cpDNA loci distribution by population.

Population	Locus	Allele (pb)	Frequency
Browsk ^1^	ccmp6	93	0.016
Hajnówka ^1^	ccmp3	95	0.016
	ccmp6	101	0.015
	ccmp7	123	0.016
	ccmp10	99	0.015
Chojnów ^2^	ccmp7	119	0.017
Kozienice	ccmp3	94	0.434
	ccmp3	98	0.013
	ccmp3	100	0.013
	ccmp7	116	0.434
	ccmp7	121	0.013
	ccmp10	97	0.013
	ccmp10	100	0.013
	ccmp10	105	0.013

^1^ Białowieża Primeval Forest; ^2^ Nature Reserve.

**Table A4:** Partitioning of genetic variation within and among the studied ash populations (AMOVA) assessed by cpDNA markers.

Source of Variability (nSSR)	d.f. ^1^	Sum of Squares	Mean Square	Estimated Share (%)	Variation	*p* Value ^2^
Among populations	4	3087.848	771.962	7.220	2%	
Within populations	276	104,623.142	379.069	379.069	98%	
Total	280	107,710.989		386.290	100%	n.a.

^1^ d.f.—degrees of freedom. ^2^ *p* value based on 999 permutations; n.a.—not available due to low genetic variability in haplotype data.

**Table A5:** Summary of private alleles of nSSR loci distribution by Vitality Degree.

Vitality Degree ^1^	Locus	Allele (pb)	Frequency
1	Femsatl-F4	218	0.018
	Femsatl-F4	222	0.018
	Femsatl-F4	234	0.009
	Femsatl-F8	131	0.018
	Femsatl-F8	185	0.027
	Femsatl-F11	198	0.009
	Femsatl-F16	190	0.021
	Femsatl-F19	164	0.009
	M2-30	260	0.009
	M2-30	272	0.009
	Femsatl-F4	208	0.008
2	Femsatl-F8	137	0.025
	Femsatl-F8	147	0.008
	Femsatl-F8	168	0.008
	Femsatl-F11	178	0.008
	Femsatl-F11	218	0.008
	Femsatl-F11	220	0.008
	Femsatl-F11	224	0.017
	Femsatl-F11	228	0.008
	Femsatl-F16	176	0.009
	Femsatl-F19	158	0.009
	Femsatl-F19	182	0.009
	Femsatl-F19	196	0.034
	Femsatl-F19	208	0.009
	M2-30	192	0.017
	M2-30	194	0.017
	M2-30	210	0.034
3	Femsatl-F4	158	0.008
	Femsatl-F4	214	0.008
	Femsatl-F4	216	0.008
	Femsatl-F8	133	0.008
	Femsatl-F11	180	0.016
	Femsatl-F11	196	0.008
	Femsatl-F11	210	0.016
	Femsatl-F16	182	0.017
	Femsatl-F19	166	0.015
	Femsatl-F19	202	0.008
	Femsatl-F19	204	0.008
	Femsatl-F19	218	0.008
	M2-30	176	0.008
	M2-30	256	0.008
	M2-30	282	0.008

^1^ after Roloff [5].

**Table A6:** Partitioning of genetic variation within and among the three degrees of vitality (AMOVA) assessed by nSSR markers.

Source of Variability (nSSR)	d.f. ^1^	Sum of Squares	Mean Square	Estimated Share (%)	Variation	*p* Value ^2^
Among populations	2	9.099	4.549	0.014	1%	
Within populations	369	1019.756	2.764	2.764	99%	
Total	371	1028.855		2.778	100%	0.001

^1^ d.f.—degrees of freedom. ^2^ *p* value based on 999 permutations.

**Table A7:** Summary of private alleles of cpDNA loci distribution by Vitality Degrees.

Vitality Degree ^1^	Locus	Allele (pb)	Frequency
1	ccmp6	100	0.017
	ccmp10	98	0.017
2	ccmp3	95	0.017
3	ccmp6	93	0.015
	ccmp6	101	0.015
	ccmp7	119	0.015
	ccmp7	123	0.015
	ccmp10	99	0.015

^1^ after Roloff [5].
